# Integrating Statistical and Machine-Learning Approaches for *Salmonella enterica* Surveillance in Northwestern Italy: A One Health Data-Driven Framework

**DOI:** 10.3390/microorganisms13122773

**Published:** 2025-12-05

**Authors:** Aitor Garcia-Vozmediano, Angelo Romano, Mattia Begovoeva, Monica Pitti, Elisabetta Crescio, Aldo Brenda, Michela Di Roberto, Anna Gioia, Adriana Giraldo, Eva Massone, Michela Nobile Lanzarini, Alessia Raggio, Erica De Vita, Giuseppe Ru, Cristiana Maurella

**Affiliations:** 1 Istituto Zooprofilattico Sperimentale del Piemonte, Liguria e Valle d’Aosta, Via Bologna 148, 10154 Turin, Italy; aitor.garciavozmediano@izsplv.it (A.G.-V.); angelo.romano@izsplv.it (A.R.); mattia.begovoeva@izsplv.it (M.B.); giuseppe.ru@izsplv.it (G.R.); 2 S.C. Sicurezza Alimentare, Istituto Zooprofilattico Sperimentale del Piemonte, Liguria e Valle d’Aosta, Via Bologna 148, 10154 Turin, Italy; 3 Centro di Riferimento per la Tipizzazione delle Salmonelle (CeRTiS), Istituto Zooprofilattico Sperimentale del Piemonte, Liguria e Valle d’Aosta, Via Bologna 148, 10154 Turin, Italy; monica.pitti@izsplv.it; 4 Instituto Tecnológico y de Estudios Superiores de Monterrey, Campus Estado de México, Av. Eugenio Garza Sada 2501 Sur, Tecnológico, Monterrey 64849, N.L., Mexico; elisabetta@tec.mx; 5 Dipartimento di Ricerca Traslazionale e delle Nuove Tecnologie in Medicina e Chirurgia, Università di Pisa, Via Savi 10, 56126 Pisa, Italy; aldo.brenda@asfo.sanita.fvg.it (A.B.); michela.diroberto@aslcaserta.it (M.D.R.); a.gioia@ausl.latina.it (A.G.); a.giraldo@asl1.liguria.it (A.G.); emassone@asl.at.it (E.M.); michela.nobile@asfo.sanita.fvg.it (M.N.L.); alessia.raggio@aslcagliari.it (A.R.); erica.devita@unipi.it (E.D.V.); 6 S.C. Sanità Animale, Azienda Sanitaria Friuli Occidentale, Via della Vecchia Ceramica 1, 33170 Pordenone, Italy; 7 Dipartimento di Prevenzione—Sanità Animale, Azienda Sanitaria Locale Caserta, Via Unità Italiana 28, 81100 Caserta, Italy; 8 Dipartimento di Prevenzione—Sanità Animale, Azienda Sanitaria Locale Latina, Via Carrara 12, 04100 Latina, Italy; 9 Dipartimento di Prevenzione—Igiene degli Alimenti di Origine Animale, Azienda Sanitaria Locale 1 Imperia, Via Aurelia Ponente 97, 18038 Bussana di Sanremo, Italy; 10 Dipartimento di Prevenzione—Sanità Animale, Azienda Sanitaria Locale Asti, Via Conte Verde 125, 14100 Asti, Italy; 11 Dipartimento di Prevenzione, S.C. Igiene e Sanità Pubblica—Epidemiologia, Azienda Socio-Sanitaria Locale di Cagliari, Piazza de Gasperi 2, 09100 Cagliari, Italy

**Keywords:** food surveillance, foodborne pathogens, XGBoost, predictive modelling, public health, One Health

## Abstract

*Salmonella enterica* is a major cause of foodborne illness globally. We analysed 41,945 food samples collected under official surveillance in Piedmont (north-western Italy) between 2013 and 2023 to characterise contamination patterns and evaluate an integrated analytical framework combining classical statistical modelling with machine-learning prediction. Overall prevalence was low (2.20%; 95% CI: 2.06–2.35) but heterogeneous across matrices, with poultry and pork displaying the highest contamination levels (11.8% and 7.14%). Risk increased at distribution/retail stages, and contamination declined markedly from 2013 to 2018, with lower levels in late autumn. Meteorological factors had minimal influence. Mixed-effects models identified food category and production stage as the main determinants of contamination, while the XGBoost algorithm showed stable predictive performance (median absolute error ≈ 0.02) and spatially coherent estimates. SHAP analyses confirmed food composition variables as the dominant predictors. These findings highlight persistent vulnerabilities within poultry and swine supply chains, particularly at post-production stages, and illustrate the complementary value of combining explanatory and predictive approaches to strengthen risk-based, One Health-aligned food-safety surveillance.

## 1. Introduction

Foodborne infections caused by *Salmonella enterica* remain a major global health and food-safety concern, generating substantial morbidity, economic losses, and social repercussions [1,2,3]. The persistence of this pathogen reflects its complex ecology, across the food chain—from farm to processing and retail—and environmental reservoirs such as soil, water, and wildlife that can re-introduce contamination into production systems [4,5,6,7,8]. These multifactorial pathways make *S. enterica* a sentinel organism for assessing hygiene and control effectiveness across agri-food sectors.

At the European and national levels, the epidemiological burden of salmonellosis shows heterogeneous but well-documented trends. Human salmonellosis declined markedly between 2008 and 2014 and subsequently stabilised at roughly twenty cases per 100,000 inhabitants, with *S.* Enteritidis, *S.* Typhimurium*,* and its monophasic variant (1,4,[5],12:i:-) accounting for most cases [9,10]. In Italy, salmonellosis remains the most frequently reported zoonosis, with 3333 confirmed cases in 2023 [10]. This epidemiological context underscores the need for integrated, multi-source surveillance systems capable of capturing variations across the food chain.

Environmental and climatic drivers further influence *Salmonella* dynamics. Increases in temperature, humidity, and rainfall have been associated with heightened bacterial survival, and with modified food-handling and storage conditions, contributing to higher contamination and infection rates [11,12,13]. In Europe, a 5–10% rise in salmonellosis incidence has been reported for every 1° C increase in mean weekly temperature above 5° C [14,15,16]. Such findings emphasise that climatic variability should be considered alongside traditional food-chain determinants when evaluating contamination risk.

Despite consolidated national surveillance systems in Italy, including Enter-Vet, which collects data on *Salmonella* isolates from animal, food, and environmental sources, and Enter-Net, which compiles enteric isolates from human cases [17,18], limited interoperability and the under-exploitation of integrated analyses continue to constrain comprehensive risk assessment [19,20,21]. The One Health framework recognises the interdependence of human, animal, and environmental health and provides the conceptual basis for integrated zoonotic-disease surveillance [22]. Yet its operational implementation remains uneven, and environmental dimensions are often underrepresented [23,24,25]. Recent international initiatives underscore the importance of cross-sectoral, interoperable surveillance systems to anticipate emerging risks [26]. Within this governance perspective, data-driven analytical frameworks are increasingly recognised for their potential to enhance early detection, optimise resource allocation, and inform control strategies [27,28,29]. Embedding predictive modelling tools within One Health surveillance thus represents not only a methodological advancement but also a strategic shift towards more anticipatory and preventive food-safety management [30,31].

However, achieving such integration in practice requires addressing the fragmentation of existing data infrastructures. Most veterinary and food-safety monitoring systems were designed primarily for compliance and reporting purposes, rather than real-time analytical interoperability [32,33]. To overcome these limitations, data pipelines across human, animal, and environmental domains must be harmonised through standardised identifiers, metadata, and open interfaces [32,34]. Such harmonisation is a key component of the broader digital transformation agenda, which is aligned with the European Data Governance Act [35] and the initiatives on data interoperability of the European Food Safety Authority (EFSA) [36]. These developments are essential to fully exploit the potential of Machine Learning (ML) and other artificial intelligence techniques within official surveillance frameworks.

Recent research has demonstrated the utility of ML approaches in food-safety surveillance, outbreak detection, and early warning systems [37,38,39,40,41,42,43,44,45,46,47,48,49,50]. However, studies that systematically combine classical inferential models with ML prediction within an operational One Health framework remain scarce, highlighting a key methodological gap and limiting translation into actionable insights for veterinary and food-safety authorities.

In this context, the present study provides a long-term, eleven-year assessment of *S. enterica* prevalence in food matrices sampled across the Piedmont region of north-western Italy (2013–2023), integrating official surveillance data with climatic variables (temperature and relative humidity). We adopted a dual analytical strategy: (i) classical statistical modelling to quantify temporal, spatial, and food-chain determinants of contamination, and (ii) predictive modelling, based on ML techniques, to evaluate predictive capacity and stability of integrated datasets. Together, these approaches were designed to address three complementary research questions:

(1) Which temporal, spatial, and food-chain factors have influenced *Salmonella* contamination in food matrices from Piedmont during the 2013–2023 surveillance period?
(2) To what extent did environmental factors—particularly temperature and relative humidity—modulate this risk within the regional context?
(3) Can integrating official surveillance and climatic data through both inferential and ML frameworks enhance contamination-risk prediction and guide targeted prevention strategies?

By answering these questions, the study aims to provide an operational model for applying data-driven analytics to foodborne surveillance within a One Health framework.

## 2. Materials and Methods

### 2.1. Data Sources and Data Management

Data on food products were retrieved from the Laboratory Information System of the *Istituto Zooprofilattico Sperimentale del Piemonte, Liguria, e Valle d’Aosta*, a public veterinary institute operating under the Italian Ministry of Health. This system systematically records all institutional, routine, and research activities related to animal health and food safety across the administrative regions under its jurisdiction (Figure S1).

An initial dataset comprising over 150,000 non-aggregated records was extracted using PL/SQL queries. Prior to analysis, data were cleaned and filtered according to predefined inclusion criteria. Only food matrices sampled within the Piedmont region and analysed under official surveillance programmes between 1 January 2013 and 31 December 2023 were retained. Sampling within these programmes follows a random design established under national and regional food-safety monitoring plans, aimed at ensuring representative coverage across food categories and production stages. Consequently, only samples collected as part of routine surveillance activities were included, whereas those obtained in response to suspected or confirmed foodborne outbreaks or epidemiological investigations were excluded. Records were further restricted to those including tests for *Salmonella* detection. Food samples were collected within the framework of official food safety surveillance and analysed in accredited institutional laboratories according to standard protocols (ISO 6579:2002, later ISO 6579-1:2017 [51,52]).

Environmental data were sourced from the Copernicus Agrometeorological Catalogue [53]. Gridded daily mean values of air temperature and relative humidity were obtained for the period 01 November 2012 to 31 December 2023 at a 0.1° × 0.1° spatial resolution, representing conditions at 2 m above ground level. Because food data were available only at a municipal level, environmental estimates were aggregated by averaging all grid-cell values whose centroids fell within each municipality, thus yielding daily municipal mean values.

Geographic coordinates of municipal centroids were retrieved from the Italian National Institute of Statistics (ISTAT) database [54]. These coordinates were used to integrate food and environmental datasets and to visualise spatial patterns of *Salmonella* occurrence.

### 2.2. Datasets and Variable Recoding

#### 2.2.1. Exploratory Data Processing

The final dataset comprised 41,945 food records, each containing information on sampling date and location (i.e., establishment, municipality, local health district), food-matrix type, animal species of origin, and test outcome.

Given the diversity of sampled matrices, a 14-level categorical variable (*food_cat*) was built, comprising the following: beverages; meat; cereals, seeds and flours; fruit; milk and dairy products; pasta; fish; composite foods/preparations; meat products; bakery and pastry products; ready-to-eat products; sauces; eggs and egg products; and vegetables.

For meat products, the variable *meat_type* grouped animal species of origin into the following six macro-categories: poultry; bovine; swine; game; other meats; and not identified. The “other meats” category included the less represented species in our dataset (i.e., equine, ovine, caprine, and rabbit), whereas the “not identified” category referred to samples lacking species information (e.g., in the case of minced products containing multiple species such as swine–bovine mixtures).

Based on establishment information, we defined a binary variable, *productive_phase*, to distinguish samples collected during production from those obtained during distribution/retail stage.

To integrate environmental data, lagged mean air temperature and relative humidity were computed for the 7, 14, 21, and 30 days preceding each sampling date. In addition, a composite Temperature-Humidity Index (THI) was calculated as an indicator of heat and humidity stress, potentially influencing bacterial persistence and contamination risk. THI was computed at daily municipal resolution using the equation proposed by Kelly and Bond [55]:

THI (°C)= *T*_air_ − 0.55 × (1 − 0.01 × RH) × (*T*_air_ − 14.5),

where *T*_air_ is the air temperature (°C) and RH is the relative humidity (%). The same lag structure was applied to THI to assess delayed combined effect of temperature and humidity on *Salmonella* contamination risk.

#### 2.2.2. Modelling and Prediction Phase Using Machine Learning (ML)

To model *Salmonella* contamination in food matrices, environmental, temporal, and compositional predictors were integrated into a unified analytical framework. Table 1 summarises the predictors considered to model *Salmonella* contamination in food.

Environmental parameters were included as potential modulators of *Salmonella* persistence and growth, since temperature and humidity jointly affect bacterial survival and transmission dynamics [56,57]. Derived indices such as THI and Vapour Pressure Deficit (VPD) were incorporated to capture combined effects of thermal and moisture stress conditions.

From the raw climatic data, THI and VPD were calculated at daily municipal resolution. VPD was derived according to the Tetens equations [58]:

*VPD* (kPa) = *es* − *ea*; *es* (mbar) = 0.6108 × *e*^(17.27×Taria)/(Taria + 237.3)^; *RH* (%) = (*ea*/*es*) × 100,

where *es* and *ea* denote the saturation and actual vapour pressure, respectively. These variables were subsequently aggregated at the municipal-month level, computing the mean temperature (*t_mean*), relative humidity (*rh_mean*), THI (*thi_mean*), and VPD (*vpd_mean*), together with the standard deviation of temperature (*t_sd*) and the number of days exceeding the THI thresholds corresponding to the 75th, 90th, and 95th percentiles of the long-term distribution (*thi_gt_n*).

To capture short-term effects, lagged versions of monthly means were generated for one and two months (*_mean_lag1*, *_mean_lag2*). To account for potential seasonality and inter-annual variability, monthly climatic anomalies were also computed as deviations from 2013 to 2020 climatological averages.

Food-surveillance data were aggregated at the same spatial-temporal resolution (municipality × month; n = 12,501 records), calculating the total number of sampled matrices, the number of *Salmonella*-positive and negative samples, and prevalence (dependent variable, expressed as a proportion).

Relative proportions of samples by production phase, food category, and meat type were standardised by the total number of tests (*share_n_variables*) and included as quantitative predictors. Geographic coordinates of municipal centroids (*long_X*, *lat_Y*) were also incorporated to account for spatial gradients.

Temporal structure was encoded to represent both seasonality and long-term trends. To model monthly seasonality and ensure continuity between December and January, Fourier terms were added (*month_sine* and *month_cosine*), while the *year* variable was centred on the median of the study period (i.e., 2019) to improve numerical stability and facilitate interpretation of temporal trends.

The resulting integrated dataset (municipality × year × month) thus contained food-surveillance indicators (number of tests, number of positive samples, prevalence, proportions by production phase, food type, and meat type), climatic variables (concurrent, lagged, and anomalous estimates), and geographic coordinates. All data processing, variable derivations, and integration procedures were performed in R 4.3.3 (R Core Team, Vienna, Austria).

### 2.3. Statistical Analyses

A two-step analytical approach was implemented: (i) regression modelling to identify statistically significant predictors, and (ii) ML analysis to enhance predictive accuracy and explore complex, non-linear relationships.

#### 2.3.1. Exploratory and Inferential Analysis

Descriptive and regression analyses of food data were conducted in Stata 17 (StataCorp, 2021, College Station, TX, USA), while feature engineering and ML analyses were performed in R 4.3.3 (R Core Team, Vienna, Austria).

During the exploratory phase, we quantified the overall prevalence of *Salmonella* contamination by food category, production phase, and meat origin, with corresponding 95% confidence intervals (CIs). The spatial and temporal distribution of surveillance activity was examined by year, month, and municipality.

To identify risk factors associated with *Salmonella* contamination in food products, univariate negative binomial regression models with robust variance were used to estimate crude effect, while accounting for overdispersion and heterogeneity in sampling intensity. Given the significant geographical variability across provinces and health districts (ASL), a multivariable generalised linear mixed model (GLMM) with a negative binomial distribution, log link function, and robust variance was fitted. The dependent variable was binary (*Salmonella*-positive vs. negative), while fixed effects included the variables food category, production phase, year and month of sampling coded as categorical variables. The ASL was included as a random effect to account for districts’ heterogeneity in surveillance activity.

The model was then extended to include environmental covariates. Temperature and relative humidity were first assessed separately to avoid collinearity, and later combined into THI, both as a single term and in interaction with sampling month to evaluate seasonal modulation. Associations were expressed as prevalence risk ratios (PRR) with 95% CIs, and statistical significance was set at *p* < 0.05.

Findings from this inferential phase guided predictor selection for the ML stage.

#### 2.3.2. Predictive Modelling Using Extreme Gradient Boosting (XGBoost)

A supervised predictive model based on Extreme Gradient Boosting (XGBoost) algorithm [59] was developed to explore non-linear relationships between food characteristics, climatic variables, and the probability of detecting *Salmonella* in food samples. Unlike traditional regression models, XGBoost enhances predictive accuracy and captures interaction effects while retaining interpretability through variable importance metrics [60]. Analyses were implemented in R 4.3.3 (R Core Team, Vienna, Austria) using the *xgboost* package (version 1.7.7.1) [61], with a fixed random seed to ensure reproducibility.

(a) ***Learning, testing, and validation strategy***

To preserve temporal independence and prevent data leakage (i.e., inclusion in the training set of information already present in the prediction set), a chronological data split was adopted. Data from 2013 to 2020 were used for model training and early stopping; data from 2021 to 2022 served as the validation set for hyperparameter tuning; and data from 2023 were retained as an independent test set for final evaluation (i.e., assessment on unseen data).

During training, a rolling-origin cross-validation scheme was implemented. Five consecutive folds were defined, using all available data up to year *t*–1 for training and data from year *t* for validation (folds corresponding to 2016, 2017, 2018, 2019, and 2020). This prospective setup simulated real forecasting scenarios and assessed temporal generalisability [62].

(b) ***Hyperparameter optimisation and final model***

Hyperparameters were optimised through random search across 40 candidate configurations (Table 2). Each configuration was evaluated via 5-fold cross-validation, using weighted Root Mean Squared Error (wRMSE)—weighted by the number of tests per municipality-month cell, the statistical unit of analysis—as the primary performance metric and Mean Absolute Error (MAE) as secondary criterion. The best-performing configuration was selected to train the final model (2013–2019), with early stopping based on 2020 data to prevent overfitting. Predictions were generated for the validation (2021–2022) and independent test (2023) datasets.

Model performance was assessed using RMSE, MAE, wRMSE, and wMAE (weighted as described above), the coefficient of determination (R^2^), and quantiles of absolute error (50th and 90th percentiles; QAE_50_ and QAE_90_). RMSE and MAE quantify the average difference between predicted and observed prevalence values, with lower values indicating more accurate predictions [63]. RMSE penalises larger errors more strongly due to its squared-error formulation, making it more sensitive to outliers, whereas MAE provides a more intuitive measure of the typical prediction error [63,64]. In practical terms, an MAE of 0.02 corresponds to an average absolute deviation of about two percentage points between predicted and observed prevalence, meaning that the model’s estimates closely track empirical values even at low prevalence levels. Likewise, an RMSE of approximately 0.03 reflects that most predictions deviate from observed values by roughly three percentage points, with higher weight given to occasional larger errors.

The weighting scheme accounts for heterogeneity in sampling intensity, giving greater influence to estimates derived from municipality-month cells with a larger number of tests.

(c) ***Model calibration***

The agreement between observed and predicted prevalence values was evaluated through logistic recalibration, regressing observed outcomes on the logit of predicted probabilities [65]. Calibration intercept (α) and slope (β) were estimated with 95% CIs, where α = 0 and β = 1 denote perfect calibration.

Calibration plots comparing observed vs. predicted prevalence across deciles of predicted risk were produced, and the weighted Expected Calibration Error (wECE) was computed using the same weighting scheme described above to quantify overall miscalibration.

(d) ***Model interpretation***

To ensure interpretability, SHapley Additive exPlanations (SHAP) were computed to quantify each variable’s additive contribution to predicted outcomes [60]. SHAP values were first calculated on the independent test set to assess local and global effects, and later on the combined 2013–2022 dataset to obtain global importance rankings. Predictor relevance was expressed as mean absolute SHAP values and visualised using beeswarm plots and dependence plots. For the most influential predictors, generalised additive models (GAMs) with penalised splines were fitted to visualise non-linear relationships and marginal effects. Temporal stability of predictor importance was evaluated by comparing SHAP rankings between 2013 and 2022 and 2023 using Spearman’s rank correlation coefficient (ρ).

**Figure 1:**
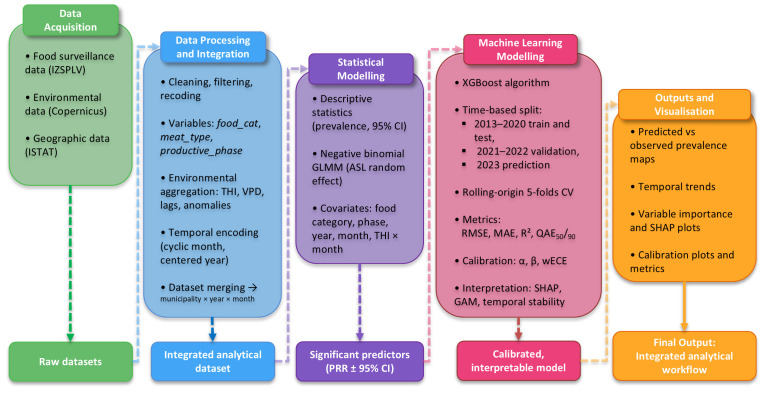
Workflow summarising data acquisition, integration, and modelling *Salmonella* contamination in food matrices sampled in Piedmont (2013–2023), encompassing classical inference and ML prediction (XGBoost), model calibration and interpretation (SHAP, GAM) and visualisation of predicted versus observed prevalence patterns at municipal level.

The full workflow—from data acquisition through modelling, calibration, and interpretation—is summarised in Figure 1.

## 3. Results

Between 2013 and 2023, a total of 41,945 food samples were analysed for *S. enterica*. The majority consisted of meat (n = 28,779; 68.6%), followed by milk and dairy products (n = 3170; 7.6%), and ready-to-eat foods (n = 3059; 7.3%), while the remaining categories—including meat products, eggs and egg derivatives, fish products, and other matrices—accounted for smaller proportions (Table 3).

Overall, *S. enterica* was detected in 2.20% of samples (95% CI: 2.06–2.35), with marked variability among food categories, except for beverages, in which no positive samples were identified (Table 3). Meat products exhibited the highest contamination levels, with a prevalence of 8.42% and a significantly greater risk (PRR = 1.99; 95% CI: 1.25–3.19) compared with raw meat (2.09%; reference PRR = 1). Within the meat and meat-product subgroups, poultry (11.8%) and swine (7.14%) displayed particularly elevated contamination levels, which translated into markedly higher relative risks (PRR = 8.85; 95% CI: 4.37–18.0 and PRR = 7.75; 95% CI: 2.82–21.3, respectively) compared with bovine meat (0.97%; reference PRR = 1). Other matrices—including milk and dairy products, eggs and egg products, sauces, bakery and pastry products, and fruit—displayed slightly lower or comparable contamination frequencies. In contrast, cereals, seeds and flours, pasta, fish, composite foods, and vegetables exhibited much lower prevalence, ranging from 0.40% to 1.58% (Table 3).

Along the agri-food chain, contamination varied significantly by sampling stage. Samples collected during the distribution/retail phase showed a prevalence of 2.99%, corresponding to a significantly higher risk of contamination (PRR = 1.88; 95% CI: 1.13–3.13) compared with those collected during production stage (Table 3).

**Figure 2:**
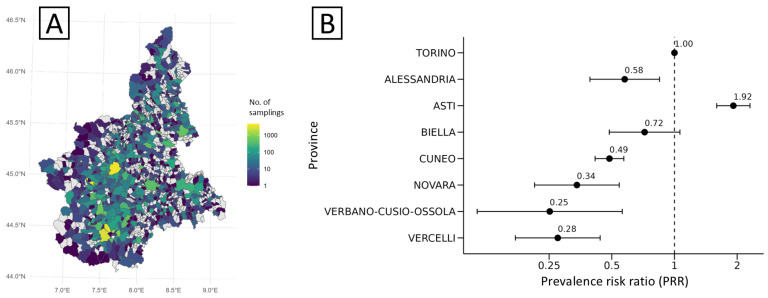
Sampling intensity (**A**) and distribution of crude risk (expressed as prevalence risk ratio, PRR) of *S. enterica* (**B**) for food matrices sampled at municipal and provincial levels, Piedmont, 2013–2023. In panel B, negative or positive deviations of point estimates (and 95% CIs) from PRR = 1 (Turin, dashed line) are statistically significant.

Geographical patterns revealed heterogeneous surveillance intensity, with denser sampling in central-southern health districts, particularly in the provinces of Cuneo and Turin (Figure 2A). The highest prevalence of *Salmonella* contamination was recorded in the province of Asti (5.56%; 95% CI: 4.40–6.54), corresponding to a significantly increased risk (PRR = 1.92; 95% CI: 1.60–2.31) compared with Turin (reference; PRR = 1; Figure 2B). Other provinces showed notably lower values, with prevalence ranging between 0.73% and 1.67%, except for Biella province (2.09%; 95% CI: 1.37–3.04), which was similar to Turin (2.90%; 95% CI: 2.65–3.17).

**Figure 3:**
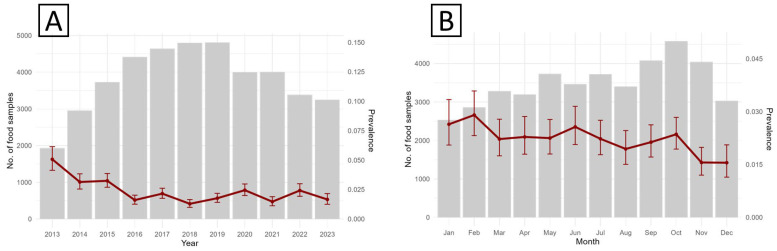
Observed annual (**A**) and seasonal (**B**) trends in surveillance activity and *S. enterica* prevalence for food matrices sampled in Piedmont, 2013–2023.

A progressive decline in contamination risk was evident over the years, particularly during 2013–2018, when the mean predicted probability of *Salmonella* contamination decreased from 4.8% (95% CI: 2.5–7.1) to 1.2% (95% CI: 0.6–2.4). Minor fluctuations were subsequently observed, with modest increases in 2020 (2.4%) and 2022 (2.3%), followed by a decline to 1.6% in 2023 (Figure 3A). Using January as the reference month (PRR = 1), no significant monthly deviations were observed (Figure 3B), except for a marked reduction in November (PRR = 0.58; 95% CI: 0.46–0.73; *p* < 0.001) and December (PRR = 0.56; 95% CI: 0.38–0.83; *p* = 0.003).

When analysed separately, the temperature and relative humidity showed no direct associations with prevalence (*p* > 0.05), including for the combined effect of both (Temperature-Humidity Index, THI) under all lag scenarios. However, during the autumn–winter period, a significant interaction between THI and December was observed (PRR = 0.89; 95% CI: 0.81–0.97), suggesting reduced contamination risk under cooler and more humid conditions.

The optimal XGBoost model exhibited consistent predictive performance across training, test, and validation sets (Table 4). Although the explained variance (R^2^) was modest, prediction errors remained relatively stable over time, as reflected by RMSE and MAE. The median absolute error (QAE50 ≈ 0.02) indicated substantial concordance between observed and predicted prevalence, while weighted errors (wRMSE, wMAE) were slightly lower, confirming improved accuracy in municipality-month cells with larger sample sizes.

**Figure 4:**
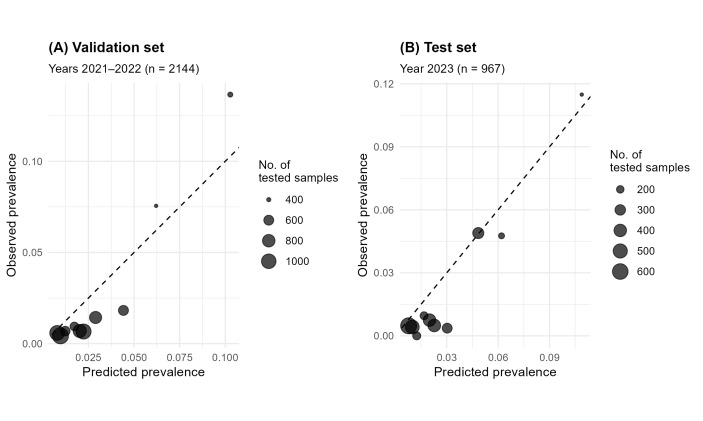
Calibration (reliability) plots for the test set (2021–2022, (**A**)) and independent validation set (2023, (**B**)). Bubbles represent the mean observed prevalence within deciles of predicted prevalence; the diagonal denotes perfect calibration. Deviations from the diagonal indicate calibration error. Bubbles size is proportional to the number of samples tested.

Calibration analysis indicated a slight systematic overestimation, reflected by negative intercepts in both test (α_test = −0.33) and validation (α_val = −0.46) sets (Table 4). Calibration slopes (β_test = 1.42; β_val = 1.28) indicated mild compression towards the mean, particularly in low-prevalence strata where predictions tended to be overestimated. Conversely, for higher prevalence values, observed and predicted estimates were closely aligned, with minimal deviations around the mean (Figure 4). Nevertheless, global calibration metrics (wECE) suggested low overall miscalibration, with an average absolute deviation of approximately 1% between observed and predicted values.

**Figure 5:**
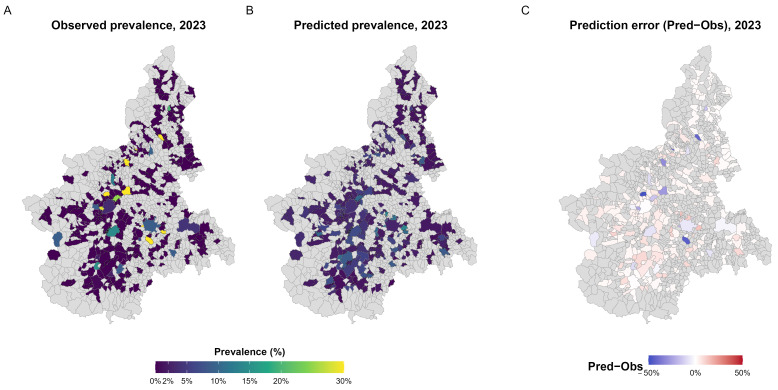
Observed, predicted, and residual prevalence of *S. enterica* in 2023. (**A**) Observed prevalence aggregated at municipal level, expressed as the proportion of positive samples among those tested. (**B**) Predicted prevalence from the XGBoost model, representing the expected probability of *S. enterica* detection in each municipality. (**C**) Prediction error (Pred−Obs) illustrating spatial deviations between predicted and observed values, with red and blue indicating model over- and under-estimation, respectively. A and B share a common colour scale (0–30%) for comparability; municipalities with fewer than 10 tested samples are outlined with dotted borders.

Spatial comparison between observed and predicted prevalence at the municipal level (Figure 5A,B) showed a coherent geographic pattern, with small residuals and no evidence of systematic spatial clustering of over- or under-estimated areas (Figure 5C).

**Figure 6:**
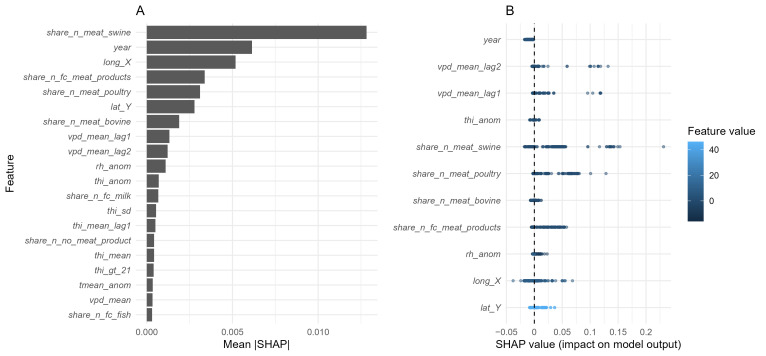
Variable importance for the XGBoost model predicting *S. enterica* positivity in food matrices collected in north-western Italy (2013–2023). (**A**) Mean absolute SHAP values illustrating the average magnitude of each predictor’s contribution to model output; (**B**) SHAP summary (“beeswarm”) plot showing the direction and magnitude of each variable’s effect: each point represents an individual observation, coloured according to the feature value (blue = low, light = high). Points located to the right of the zero line increase the predicted risk, whereas those to the left decrease it.

Among the forty-five candidate predictors, the dominant determinants of predicted *Salmonella* prevalence were related to food matrix composition, followed by temporal and spatial factors (Figure 6A,B). These associations were stable over time, as indicated by a high correlation (Spearman’s ρ = 0.959) between variable importance rankings from 2013 to 2022 and 2023.

**Figure 7:**
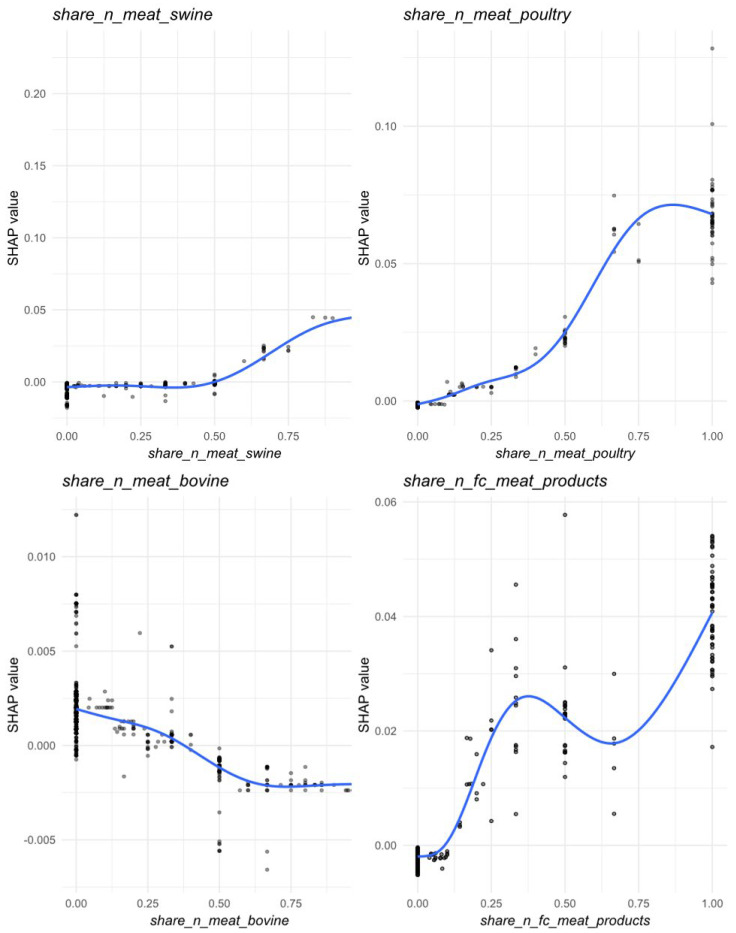
Partial dependence (marginal) effects for the food categories most contributed in the prediction of *Salmonella* prevalence across Piedmont region, 2023. Circles represent individual SHAP values; darker shading indicates greater overlap of observations due to point transparency. The blue curve is a spline smoother (GAM), summarizing the average relationship between the feature value and its SHAP contribution.

Within food categories, the proportion of swine meat samples (*share_n_meat_swine*) emerged as the most influential predictor, showing marked increases in SHAP values when this category represented ≥75% samples per municipality-month cell (Figure 7). Poultry meat (*share_n_meat_poultry*) also exhibited a positive contribution to *Salmonella* prevalence, which gradually flattened once poultry became the dominant matrix within a sampling unit. Meat products (*share_n_fc_meat_products*) displayed a non-linear association, with increased SHAP values when they represented either a minor or predominant proportion of samples, suggesting variable contamination risk along the processing chain. Conversely, bovine meat (*share_n_meat_bovino*) showed a modest but consistently negative contribution, indicating slightly lower predicted prevalence in sampling units dominated by this matrix (Figure 6B and Figure 7).

**Figure 8:**
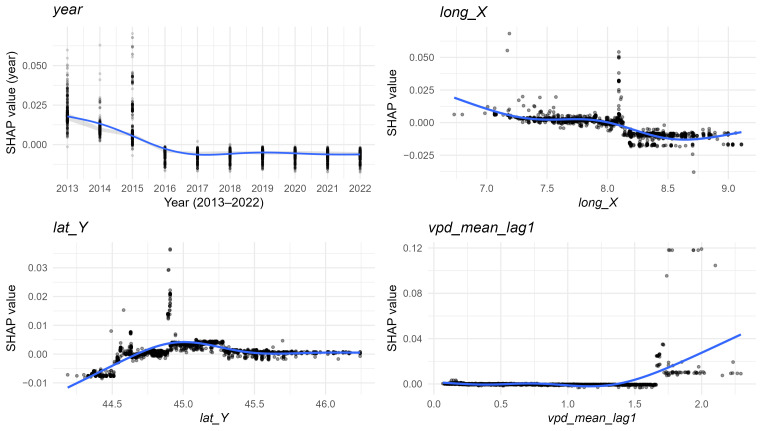
Partial dependence plots of temporal, geographic, and climatic variables that most contributed to the prediction of *Salmonella* prevalence across Piedmont region, 2023. Circles represent individual SHAP values; darker shading indicates greater overlap of observations due to point transparency. The blue curve is a spline smoother (GAM), summarizing the average relationship between the feature value and its SHAP contribution.

Among temporal variables, centred year (*year*) confirmed a progressive long-term decline in prevalence (Figure 6B and Figure 8). Spatial variables, represented by municipal centroid coordinates (*lat_Y*, *lon_X*), indicated spatial heterogeneity in *Salmonella* distribution as follows: latitude peaked around 45°, while longitude suggested a decreasing eastward gradient, with slightly higher predicted values in the western provinces (Figure 8).

Climatic variables, although less influential, still contributed marginally to model predictions (Figure 6A,B). Relative humidity anomalies (*rh_anom*) displayed a non-linear association, with positive SHAP values under moderately negative anomalies (≈ −10 to −5), indicating increased predicted prevalence during drier-than-average conditions, and negative contributions under wetter anomalies (Figure 8). Temperature-Humidity Index anomalies (*thi_anom*) exerted minimal influence, showing only a slight negative effect under warmer-than-average conditions. Lag VPD mean (*vpd_mean_lag1*) showed a slight positive effect at higher values, suggesting marginally increased predicted prevalence under drier atmospheric conditions, whereas mean temperature anomalies (*t_mean_anom*) contributed negligibly.

## 4. Discussion

This study provides a comprehensive assessment of the prevalence of *S. enterica* in food matrices sampled in Piedmont between 2013 and 2023, using an integrated analytical framework that combines classical statistical approaches with ML techniques. By bridging descriptive, inferential, and predictive analyses, our study delineates critical points of vulnerability along the agri-food continuum, while testing the feasibility of integrating diverse data streams under a One Health perspective.

Poultry and swine were confirmed as the matrices at highest risk, consistent with previous evidence identifying these production chains as major reservoirs of *Salmonella* [66,67]. The increased frequency of contamination in meat products compared with raw meat likely reflects cross-contamination during processing, handling, or equipment sanitation [68,69,70,71], highlighting the need for targeted hygiene controls during transformation steps. Similarly, the increased risk detected at the distribution and retail stages underscores the importance of maintaining strict post-production hygiene measures and ensuring cold-chain integrity, where microbial amplification and cross-contamination are most likely to occur [72,73,74].

From a spatial perspective, the pronounced heterogeneity across provinces—particularly the elevated prevalence recorded in Asti—may reflect structural differences in local supply chains, livestock density, and production practices, as reported for other Italian and European settings [10,75,76]. Conversely, higher sampling intensity in Cuneo and Turin may have contributed to differences in prevalence through surveillance bias, a limitation also recognised in comparable monitoring programmes [77,78]. Temporally, the marked decline in contamination risk from 2013 to 2018, followed by relative stabilisation, mirrors the broader European downward trend in *Salmonella* prevalence [79,80]. This decrease may be attributed to improvements in biosecurity standards, the implementation of EU-wide control programmes, and strengthened food safety management systems across animal production chains [81,82,83]. The reduction observed in November and December suggests a potential seasonal pattern, although climatic factors appeared to exert only a limited influence overall.

Neither temperature nor relative humidity showed direct associations with *Salmonella* prevalence, and the THI exhibited only a marginal effect, primarily under drier winter conditions. This limited influence contrasts several European reports that associate meteorological conditions—particularly extreme weather conditions—with increased foodborne disease incidence in humans [13,84,85,86]. Such differences likely stem from the distinct nature of the data: while human case notifications reflect exposure pathways strongly modulated by ambient climate and behavioural factors, our dataset represents post-harvest food environments where processing, transport, and storage take place under controlled conditions that markedly buffer external temperature and humidity. Consequently, the direct influence of outdoor climate on contamination risk is expected to be substantially attenuated within refrigerated or regulated food-chain settings. Moreover, the climatic data available for this study were aggregated at municipal and monthly scales, which may have masked short-term temperature spikes, humidity extremes, or microclimatic variability known to influence *Salmonella* survival [87,88,89]. Further research should therefore consider high-resolution meteorological data (e.g., daily extremes, facility-level microclimate) and exposure metrics aligned more precisely with sampling operations to improve detection of environmentally driven effects.

The application of the XGBoost algorithm provided an additional predictive dimension to the analytical framework. Although the explained variance was modest, the model demonstrated stable predictive performance across all phases—training, validation, and test—with low absolute errors and satisfactory calibration. This suggests that the algorithm effectively captured the main sources of variability in *Salmonella* prevalence, despite inherent noise in surveillance data. Spatial mapping of the observed and predicted prevalence further confirmed the consistency of model outputs, showing close agreement across most municipalities and no systematic spatial bias in prediction errors. Recent studies corroborate the utility of ML approaches, particularly gradient-boosting models such as XGBoost, for outbreak forecasting, pathogen source attribution, and risk stratification in foodborne surveillance [38,39,45,90,91]. Operationally, systems such as FINDER illustrate how ML algorithms applied to unconventional data sources (e.g., geolocated web searches) can enhance early detection of at-risk food environments, demonstrating their potential for large-scale, heterogeneous surveillance datasets [46,92].

Compared with traditional statistical approaches, which are constrained by assumptions of linearity, independence, and limited capacity to handle high-dimensional or correlated data [93,94], ML algorithms such as XGBoost offer enhanced flexibility to capture non-linear relationships, high-order interactions, and complex interactions among variables without the need for a priori specification [95,96]. This capability is particularly valuable in food-safety surveillance, where heterogeneous data sources—microbiological, climatic, spatial, and temporal—often violate the assumptions of linearity and independence underpinning classical statistical frameworks. By modelling complex dependencies and non-linear interactions, XGBoost enhances predictive performance in such heterogeneous environments while maintaining robustness against noise [97]. Nevertheless, these approaches should be viewed as complementary rather than substitutive to classical inferential models, which remain essential for hypothesis-driven analysis and causal interpretation. A concise comparative summary of the complementary roles of these two methodological approaches is provided in Table S1. The integration of both explanatory and predictive perspectives therefore provides a more comprehensive understanding of *Salmonella* contamination dynamics within a One Health framework.

To ensure transparency and interpretability—critical prerequisites for the operational credibility of ML models in public-health contexts [98,99]—we applied SHAP values to quantify the contribution of each predictor and mitigate the “black-box” limitation commonly attributed to ensemble methods [60,97]. In this study, SHAP analyses revealed that the composition of sampled food categories—particularly the proportions of swine and poultry—were the most influential determinants of *Salmonella* prevalence, followed by spatial and temporal predictors, whereas climatic factors contributed minimally. Complementary interpretability tools, such as Generalised Additive Models (GAMs), further allowed the visualisation of marginal and non-linear effects, transforming complex algorithmic relationships into epidemiologically meaningful insights [100]. Embedding these interpretability and calibration analyses within predictive workflows not only strengthens scientific accountability but also enhances the translation of analytical outputs into policy-relevant evidence for surveillance and risk management. The convergence of findings from both classical and ML approaches reinforces the internal consistency of our results and highlights the complementary nature of explanatory and predictive modelling in advancing data-driven One Health surveillance.

The main strengths of this study include (i) a long-term dataset spanning eleven years of harmonised official surveillance, ensuring high laboratory comparability; (ii) the integration of microbiological, environmental, and geographic information within a unified analytical framework; (iii) rigorous temporal validation to prevent data leakage and overfitting; and (iv) explicit calibration and interpretability assessments (SHAP, GAM) to ensure model transparency. Nonetheless, several limitations must be acknowledged. The observational design of surveillance data limits causal inference; uneven sampling intensity across provinces may have introduced detection bias; and the ecological and temporal aggregation of climatic variables (municipal-monthly) may have attenuated short-term or micro-scale environmental effects. Moreover, unmeasured confounders, such as hygiene standards within establishments or transport conditions, may have influenced contamination risk. Additional sources of potential bias should also be considered. First, outbreak-related samples were excluded because they originate from targeted, non-random investigations and would have inflated contamination estimates, thereby reducing the representativeness of baseline surveillance patterns. Second, the representativeness of food matrices was uneven, with meat, dairy, and ready-to-eat products oversampled relative to other categories, reflecting regulatory priorities; this imbalance may have affected the precision of prevalence estimates and the relative importance attributed to specific predictors. Third, human *Salmonella* data were not integrated, as the focus of this work was the predictive and explanatory performance of food surveillance datasets; although integration with human epidemiological information is valuable and has been explored in a previous study [45], differences in surveillance structures and temporal scales placed such linkage beyond the scope of the current analysis. Finally, the modest proportion of explained variance, despite good absolute error control, suggests residual heterogeneity that merits further exploration.

From a veterinary public health perspective, these findings underscore the importance of prioritising risk management measures within poultry and swine supply chains and reinforcing hygiene controls at the post-production and retail stages, where cross-contamination risks are highest. The integration of predictive mapping within surveillance workflows could enhance situational awareness and facilitate the visual identification of areas with higher predicted contamination risk, supporting data-driven resource allocation and inspection planning [101,102]. More broadly, the combined use of explanatory (GLMM) and predictive (XGBoost) modelling represent a pragmatic framework for risk assessment, balancing interpretability with operational applicability. Future research should focus on linking pathogen genomic data with environmental and food-chain predictors, exploring the potential of near-real-time data integration for early warning and adaptive surveillance. Such advances could substantially enhance the capacity of official monitoring systems to anticipate emerging risks and support evidence-based decision-making in food safety management.

## 5. Conclusions

In this study, the prevalence of *S. enterica* in food matrices from Piedmont was mainly driven by food-category composition, supply chain stage, and spatial–temporal factors, whereas the effect of the environmental conditions, limited here to temperature and humidity, appeared marginal. These results emphasise the importance of risk-based and continuous monitoring across the agri-food chain—particularly within poultry and swine sectors—and the need for stringent hygiene controls during post-production and distribution stages.

Methodologically, the combined use of classical statistical and ML models proved feasible and informative for integrating microbiological, climatic, and geographical information within a unified predictive framework. Despite inherent limitations related to surveillance-system design and the coarse resolution of environmental data, the models exhibited stable performance and satisfactory calibration, highlighting the potential of advanced predictive tools to enhance accuracy and interpretability in operational food-safety surveillance.

Building on the present framework, the integration of ML tools with multisource and interoperable datasets may substantially strengthen the epidemiological monitoring of foodborne zoonoses in alignment with the One Health paradigm. This will require external validation across different geographical and production systems, finer spatial and temporal data granularity, and a deeper assessment of environmental and facility-level determinants, including extreme weather events and hygiene management practices.

In summary, this study demonstrates that integrating explanatory and predictive analytical strategies can enhance prevention and control of foodborne pathogens and, more broadly, inform evidence-based governance within increasingly complex agri-food and environmental systems.

## Figures and Tables

**Table 1:** List and description of predictors incorporated in the ML model (XGBoost) for estimating *Salmonella* contamination probability.

Variable	Description	Definition
*year*	Centred year	Sampling year centred on 2018. It ranges from –5 (2013) to +5 (2023).
*month_sine,* *month_cosine*	Cyclic month encoding	Sine and cosine transformations of month to represent annual periodicity.
*long_X,* *lat_Y*	Geographic coordinates	Longitude and latitude of the sampling municipality centroid.
*share_production,* *share_retail*	Sampling-phase composition	Proportion of samples collected in production vs. distribution/retail.
*share_n_meat_species*	Meat-type composition	Proportion of meat samples belonging to each species category (swine, poultry, bovine, game, other, none). One variable per species.
*share_n_fc_food_cat*	Food-category composition	Proportion of samples in each processed food category (e.g., cured meats, vegetables, fruit, bakery, etc.). One variable per category.
*_anom	Climatic anomalies	Difference between monthly observed values and 2013–2020 climatological means * One variable per parameter: *t_mean, thi_mean, vpd_mean*.
*thi_mean*	Mean THI	Monthly mean Temperature-Humidity Index (°C) derived from temperature and humidity.
*thi_sd*	THI variability	Standard deviation of THI (°C) during the sampling month.
*vpd_mean*	Mean VPD	Monthly mean Vapour Pressure Deficit (kPa).
*thi/vpd_mean_lag1, thi/vpd_mean_lag2*	Lagged climatic means	Monthly means of THI or VPD lagged by one or two months relative to sampling.
*thi_gt_percentile*	THI exceedance days	Number of days exceeding the 75th, 90th, and 95th percentiles of the long-term THI distribution. One variable per percentile.

**Table 2:** Search space for XGBoost hyperparameters (random search, 40 candidates). For each hyperparameter, the distribution and sampling range are reported.

Parameter	Description	Distribution	Range/Values
*max_depth*	Maximum tree depth	Discrete	{3, 4, 5, 6, 7, 8}
*eta*	Learning rate	Log-uniform	[0.02, 0.20]
*min_child_weight*	Minimum child node weight	Discrete	{1, 3, 5, 10}
*subsample*	Subsample ratio	Uniform	[0.60, 0.90]
*colsample_bytree*	Column subsampling per tree	Uniform	[0.60, 0.90]
*gamma*	Minimum loss reduction per split	Uniform	[0.60, 0.90]

**Table 3:** Prevalence of *Salmonella enterica* in food matrices sampled in Piedmont, 2013–2023. Estimates are reported by food category, animal species of origin (for meat products), production phase, province, and local health district (ASL).

	No. Tested Samples	No. of Positives	Prevalence (%) [95% IC]	PRR [95%IC]	*p*
***Food category***					
Beverages	174	0	0 [0–2.1]	-	-
Meat	28,779	602	2.09 [1.93–2.26]	1 (Ref.)	-
Cereals, seeds, and flours	593	6	1.01 [0.37–2.19]	0.29 [0.12–0.71]	<0.01
Fruit	169	5	2.96 [0.97–6.77]	0.92 [0.38–2.22]	
Milk and dairy products	3170	75	2.37 [1.87–2.96]	0.79 [0.47–1.31]	
Pasta	190	3	1.58 [0.33–4.54]	0.36 [0.15–0.83]	<0.05
Fish products	1115	19	1.70 [1.03–2.65]	0.45 [0.22–0.90]	<0.05
Food preparations	676	2	0.30 [0.04–1.06]	0.08 [0.02–0.30]	<0.001
Meat products	1426	120	8.42 [7.03–9.98]	1.99 [1.25–3.19]	<0.01
Bakery and pastry products	476	7	1.47 [0.59–3.01]	0.40 [0.14–1.11]	
Ready-to-eat products	3059	48	1.57 [1.16–2.08]	0.34 [0.22–0.53]	<0.001
Sauces	175	7	4.0 [1.62–8.07]	0.76 [0.35–1.65]	
Eggs and egg products	1148	18	1.57 [0.93–2.47]	0.91 [0.37–2.25]	
Vegetables	795	11	1.38 [0.69–2.46]	0.30 [0.17–0.55]	<0.001
***Origin of meat products***					
Bovine	22,869	221	0.97 [0.84–1.10]	1 (Ref.)	
Poultry	1139	134	11.8 [9.95–13.8]	8.85 [4.37–18.0]	<0.001
Swine	4311	308	7.14 [6.39–7.95]	7.75 [2.82–21.3]	<0.001
Game	894	3	0.34 [0.07–7.95]	0.37 [0.12–1.19]	
Mixed or unidentified	1130	62	5.80 [4.48–7.38]	3.21 [1.25–8.27]	<0.05
Other meats	339	4	1.18 [0.32–3.0]	0.84 [0.36–1.96]	
***Productive phase***					
Production	29,791	560	1.88 [1.73–2.04]	1 (Ref.)	
Distribution/retail	12,154	363	2.99 [2.69–3.30]	1.88 [1.13–3.13]	<0.05

Note: Categories with PRR = 1 (Ref.) were used as the reference level within each variable in the regression model.

**Table 4:** Performance and calibration metrics for the XGBoost model across training, test, and validation sets.

*Model performance*							
Set	RMSE	MAE	wRMSE	wMAE	R^2^	QAE50	QAE90
Training (2013–2020)	0.140	0.054	0.093	0.037	0.094	0.024	0.076
Validation (2021–2022)	0.120	0.048	0.091	0.036	0.031	0.021	0.071
Test (2023)	0.134	0.052	0.087	0.033	0.009	0.021	0.069
***Model calibration***							
**Set**	**Intercept (α)** **(95% IC)**		**Slope (β)** **(95% IC)**		**wECE**	**Max. Absolute** **Gap**	
Validation (2021–2022)	−0.33 (−0.50–−0.16)		1.42 (1.23–1.61)		0.012	0.034	
Test (2023)	−0.46 (−0.73–−0.19)		1.28 (0.99–1.58)		0.01	0.027	

Note: Weighted performance and calibration metrics (wRMSE, wMAE, wECE) were computed using as weights the number of tests performed per municipality-month cell (the statistical unit of analysis). This approach accounts for heterogeneity in sampling intensity, assigning greater influence to estimates derived from cells with larger sample sizes.

## Data Availability

The original contributions presented in this study are included in the article/Supplementary Materials. Further inquiries can be directed to the corresponding author.

## Supplementary Materials

The following supporting information can be downloaded at https://www.mdpi.com/article/10.3390/microorganisms13122773/s1, Figure S1: Geographical context of the study area. Left: Administrative provinces of the Piedmont region (Italy) with provincial boundaries and labels. Right: Map of Italy highlighting the regions under the jurisdiction of the *Istituto Zooprofilattico Sperimentale del Piemonte, Liguria, e Valle d’Aosta*. Table S1: Comparative summary of statistical and ML methods applied in this study.
