# Supplementary material for: Integrating Statistical and Machine-Learning Approaches for Salmonella enterica Surveillance in Northwestern Italy: A One Health Data-Driven Framework
Source: Microorganisms. 2025 Dec 5;13(12):2773. doi: 10.3390/microorganisms13122773 (PMC12735743; doi:10.3390/microorganisms13122773)
Supplement: Supplementary file 1 [file microorganisms-13-02773-s001.zip › microorganisms-4006579-supplementary.pdf]

Supplementary Materials

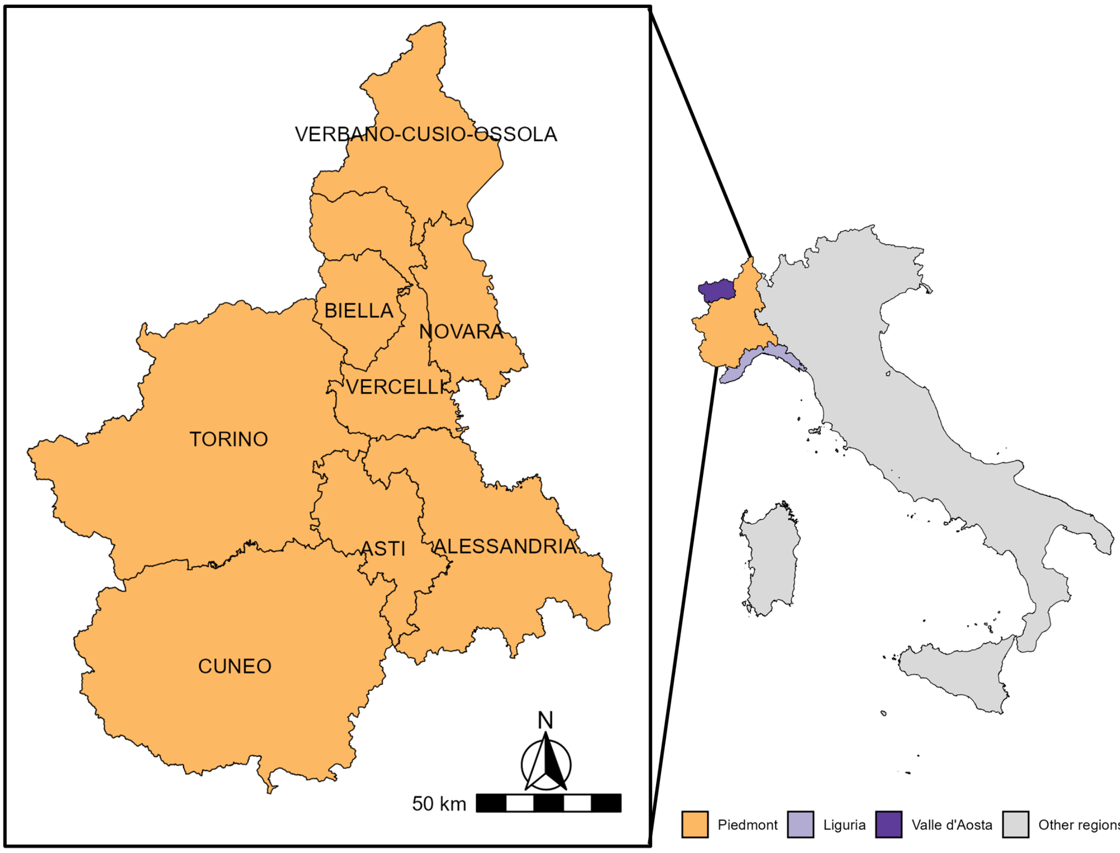

Figure S1. Geographical context of the study area. Left: Administrative provinces of the Piedmont region (Italy) with provincial boundaries and labels. Right: Map of Italy highlighting the regions under the jurisdiction of the *Istituto Zooprofilattico Sperimentale del Piemonte, Liguria, e Valle d'Aosta*.

Table S1. Comparative summary of statistical and ML methods applied in this study.

Methodological Dimension	Statistical Modelling	Machine-Learning Modelling
**Analytical objective**	Hypothesis-driven inference	Pattern discovery and prediction
**Interpretability**	High–effect estimates directly interpretable	Moderate to low–model structure less transparent
**Model assumptions**	Requires predefined functional form; linearity on link scale	Non-parametric; no assumption on data structure
**Handling of non-linearity and interactions**	Limited unless explicitly modelled	Excellent–automatically captures interactions and non-linear patterns
**Risk of overfitting**	Lower due to contrained model structure	Higher without proper tuning and validation
**Primary outputs**	Effect sizes, uncertainty measures, significance	Variable importance, predictive ranking, partial dependence
**Biological insight**	Strong for quantifying associations	Useful for identifying influential predictors
**Recommended use**	For interpretative and epidemiological decision-making	Exploratory analysis (i.e., pattern recognisition) and enhancement of surveillance prediction
